# Behaviour and associated determinants of COVID-19 vaccine acceptance and advocacy: a nationwide survey of pharmacy professionals in Qatar

**DOI:** 10.1186/s40545-023-00668-4

**Published:** 2023-11-28

**Authors:** Moza Al Hail, Pallivalapila Abdulrouf, Derek Stewart, Wessam Elkassem, Rajvir Singh, Rasha Al Enany, Aftab Mohammed Azad, Asma Tarannum, Binny Thomas

**Affiliations:** 1 Pharmacy Executive Directors Office, Hamad Medical Corporation, Doha, Qatar; 2 College of Pharmacy, Qatar University, Doha, Qatar; 3 Department of Pharmacy, Al Wakra Hospital, Doha, Qatar; 4 Corporate Department of Emergency Medicine, Hamad Medical Corporation, Doha, Qatar; 5 St John of God, Midland Public and Private Hospitals, Perth, Australia; 6 Cardiology Research, Adult Cardiology Dept, Heart Hospital, Doha, Qatar

**Keywords:** COVID-19, Vaccines, Hesitancy, Vaccine acceptance, Theory, Behavior, Qatar

## Abstract

**Background:**

Vaccine hesitancy poses a global challenge and is acknowledged to be a complex, multifactorial phenomenon. Of particular concern is hesitancy among health professionals, as this may also impact their advocacy roles. There is a lack of theory-based investigations of pharmacy professionals.

**Aim:**

The study aims to determine the behaviour and associated determinants influencing pharmacy professionals’ attitude towards vaccine acceptance and advocacy.

**Methods:**

A cross-sectional survey of 2400 pharmacists and pharmacy technicians at government, semi-government, and private community pharmacies in Qatar. Questionnaire items captured perspectives on COVID vaccine acceptance, advocacy and associated determinants based on the domains and constructs of the Theoretical Domains Framework (TDF). Data were analysed by descriptive and inferential statistics, with TDF items subjected to principal components analysis (PCA).

**Findings:**

The response rate was 38.6% (927/2400). Almost all (n = 825, 89.0%) were willing to receive the vaccine, which was higher for males (p < 0.001) and those in polyclinics (p < 0.05). PCA of acceptance items gave five components, with response to ‘emotions’ being most negative, associated with acceptance (p < 0.001) and more negative in females (p < 0.001). The majority (n = 799, 86.2%) agreed that it was their professional duty to advocate vaccines. PCA for advocacy items gave two components, with the most negative responses for ‘professional role and identity’, which were more negative for those working in hospitals (p < 0.05).

**Conclusion:**

Respondents were least positive regarding emotion-related behavioral determinants for acceptance and professional role and identity for advocacy. Behavior change technique interventions that target these issues have the potential to influence the vaccine hesitancy of pharmacy professionals and other individuals.

## Background

COVID-19 continues to impose a substantial burden on healthcare [1–3], with figures as of January 2023 being in excess of 692 million cases and 6.9 million deaths worldwide [4]. Achieving herd immunity through mass immunization remains the most efficient and cost-effective health intervention [5]. Despite a multitude of public health initiatives to improve vaccination rates, hesitancy remains a major global challenge [6, 7]. The World Health Organization Strategic Advisory Group of Experts on Immunization defines vaccine hesitancy as a ‘delay in acceptance or refusal of a vaccine despite its availability’ [8]. Hesitancy is a multifactorial phenomenon often interwoven with psychological and behavioral factors, including misinformation, past negative experiences, and safety concerns [9, 10]. The behavior of vaccine hesitant individuals is complex and may vary widely due to contextual factors (religious belief, social norms, trust in vaccine providers, political or economic factors), individual or group influences (self-perception, influences of family and friends), or vaccine-related issues (safety and efficacy) [11]. Of particular concern is hesitancy among health professionals, with evidence from systematic reviews revealing inconsistent vaccine acceptance rates of 28–73% among different health professional groups [12–15].

Given the complexity of changing behaviour, The United Kingdom(UK) Medical Research Council (MRC) framework for developing and evaluating complex interventions advocates the use of theories for practice change interventions [16]. Michie et al. consolidated [17–20] 33 psychological theories of behavior and 128 theoretical constructs to synthesize 14 behavioral domains, forming the Theoretical Domains Framework (TDF). A rapid review by Crawshaw et al. [12] included 74 studies investigating healthcare professionals’ hesitancy toward the COVID-19 vaccine, with the findings of each study mapped to TDF. Approximately 35% of healthcare professional workers were vaccine hesitant, with the most common behavioral determinants being ‘beliefs about consequences’, ‘social/professional role and identity’ and ‘social influences. Furthermore, the review demonstrated a paucity of original theory-based research investigating vaccine behavior and associated determinants.

Vaccine hesitancy by health professionals may also impact their roles in vaccine advocacy, defined as the ‘promotion of the best scientific knowledge, moral attitudes, and public health practice with regard to vaccination’ [21]. The accessibility of pharmacists and pharmacy technicians heightens their roles in vaccine advocacy and increasingly in vaccine administration [22]. This role is generally well respected and considered a reliable source of information [23–26], with studies demonstrating the impact of higher vaccine acceptance rates [27]. Conversely, those eliciting hesitancy or hesitant attitudes are less likely to advocate vaccines, amplifying poor uptake [23]. A recent systematic review of vaccine advocacy by pharmacists among 25 low-middle income countries demonstrated increased vaccine confidence among the general public [25]. Of the studies in the review by Crawshaw et al., only one study explicitly reported data from pharmacists and did not include the wider pharmacy team of pharmacy technicians. In addition, only a few studies were reported from the Middle East. Anecdotal evidence suggests that due to cultural, social or religious factors, a growing proportion of the Middle Eastern population still perceives vaccination as unsafe or unwarranted [28–30].

Despite the critical importance of vaccination in public health, there is a paucity of theory driven studies understanding the factors influencing vaccine-related behaviours. The use of theory based studies will allow comprehensive exploration of vaccination behavior and the development of evidence based strategies promoting vaccine acceptance and mitigating vaccine hesitancy [17, 18, 31]. The current study therefore aims to determine pharmacy professionals’ behaviour and the associated determinants of COVID-19 vaccine acceptance and advocacy.

## Methods

### Study design and setting

A cross-sectional survey was conducted across all government, semigovernment, and private pharmacies in Qatar. Most pharmacists practicing in Qatar are expatriates predominantly from India, Egypt, Sudan or Jordan. There are over 400 community pharmacies, and pharmacies associated to 31 primary healthcare centres, 12 hospitals managed by Hamad Medical Corporation spread across different geographical locations in Qatar.

### Questionnaire development

The draft questionnaire was based on relevant literature with items in the domains of vaccine acceptance and vaccine advocacy behaviour, related behavioral determinants, and demographics and practice characteristics [32–35]. Behavioral determinant items were derived from TDF, based on the Determinants of Implementation Behavior Questionnaire [19], measured using 5-point Likert scales (strongly agree to strongly disagree). The questionnaire also contained questions specifically related to ‘willingness’ to receive the COVID-19 vaccine and its advocacy. The draft questionnaire was reviewed for face and content validity by a panel of 5 academics, researchers and practicing pharmacists with expertise in questionnaire development and use of TDF. This was followed by piloting in a convenience sample of 80 pharmacy professionals and test–retest reliability of Likert scale items at an interval of 15 days. Data from pilot participants were excluded from the final analysis.

### Recruitment and data collection

At the time of the study, 2400 practicing pharmacists and pharmacy technicians were registered with the Department of Healthcare Professions (DHP) at the Ministry of Public Health, Qatar [36] were eligible to participate. The questionnaire was distributed via email and professional WhatsApp groups in April 2021, with three reminders sent at weekly intervals. Detailed information about the study was provided, and anonymity was assured to encourage response.

### Statistical analysis

Data were analysed using SPSS v25, comprising descriptive and inferential statistics. The relationship between demographic variables (gender, age, profession, years of experience, etc.) and vaccine behavior (previous vaccination history, willingness to vaccinate, willingness to advocate) was tested using the chi-square test; p < 0.05 was considered significant. Indices for behavioral determinants of acceptance and advocacy were calculated by assigning numerical values of -2 to 2 to Likert scale responses (− 2, strongly disagree; − 1, disagree; 0, neutral; 1, agree; 2, strongly agree), with negative items reverse coded. Percentage values for each index were calculated, with zero representing overall neutrality. Internal consistency was measured using Cronbach’s alpha, with greater than 0.6 considered acceptable [37]. Relationships between demographic variables and index scores were determined using independent samples t tests and one-way ANOVA.

To reduce the large number of behavioral determinant items to a smaller number of components, these were subjected to principal component analysis (PCA). Factor retention was based on the meaningfulness of the results, visual inspection of the scree plot and eigenvalues > 1. The Kaiser–Meyer–Olkin (KMO) measure of sampling adequacy and Bartlett’s test of sphericity were used to assess the suitability of data for factor analysis [38, 39]. This included items that were not freestanding, had a correlation coefficient > 0.5 and had a high internal reliability > 0.6. [37] Orthogonal varimax rotation was performed to interpret the components [40]. Relationships between demographic variables and individual components were tested using independent t tests and one-way ANOVA.

## Results

### Demographic and practice characteristics

Nine hundred and twenty-seven completed questionnaires were received (response rate of 927/2400; 38.6%), 771 from pharmacists (83.2%) and 156 from pharmacy technicians (16.8%). Most respondents were aged between 25–34 years (n = 466, 50.3%), predominantly male (n = 531, 57.3%), and employed as hospital pharmacists (n = 493, 53.2%) (Table 1).

**Table 1:** Demographics and practice characteristics (n = 927)

Variable (n = 927)	Category	Frequency (%)
Gender	Male	531 (57.3)
	Female	396 (42.7)
Age (years)	< 25	27 (2.9)
	25–34	466 (50.3)
	35–44	319 (34.4)
	45–60	109 (11.8)
	> 60	6 (0.6)
Place of origin (based on World Health Organization regions)	Eastern Mediterranean	425 (45.8)
	South-East Asia	409 (44.1)
	Western Pacific	50 (5.4)
	The Americas	22 (2.4)
	African	13 (1.4)
	European	8 (0.9)
Place of receiving entry to practice degree	Eastern Mediterranean	511 (55.1)
	South-East Asia	349 (37.6)
	Western Pacific	28 (3.0)
	The Americas	17 (1.8)
	European	11 (1.2)
	Other	11 (1.2)
Highest Academic Qualification	Bachelor or equivalent	456 (49.2)
	Master or equivalent	172 (18.6)
	Diploma	165 (17.8)
	Doctor of Pharmacy	96 (10.4)
	Graduate Certificate of Diploma	34 (3.7)
	PhD	4 (0.4)
Duration licensed in Qatar (years)	< 1	30 (3.2)
	1–5	242 (26.1)
	6–10	177 (19.1)
	11–15	258 (27.8)
	> 15	220 (23.7)
Employed at	Hospital	493 (53.2)
	Community pharmacy	383 (41.3)
	Polyclinic/Dental clinic	51 (5.5)
Role	Staff pharmacist	459 (49.5)
	Pharmacy supervisor/manager	218 (23.5)
	Pharmacy technician	156 (16.8)
	Clinical Pharmacist/specialist	94 (10.1)

### COVID-19 vaccine acceptance behavior and behavioral determinants

At the time of the study, 556 respondents (60.0%) had received the COVID-19 vaccine, and almost all (n = 825, 89.0%) were willing to receive the vaccine. Just over half (n = 508, 54.8%) had received the seasonal influenza vaccine in the last 3 years, and 139 (15.0%) had refused vaccination in the last 3 years. The willingness to receive the COVID-19 vaccine was higher among males than females (492 (92.7%) vs 333 (84.1%), p < 0.001) and for those in polyclinics than those in hospitals and communities (51 (100%) vs 439 (89.0%) vs 335 (87.5%), p < 0.05). Those previously accepting any vaccination had a higher willingness for COVID-19 vaccination than those previously refusing (719 (91.2%) vs 106 (76.3%), p < 0.001).

The mean percentage (standard deviation) of behavioral determinant index for COVID vaccine acceptance was 31.2 ± 19.6 (scale -100 to 100), with an internal consistency of 0.71. Index scores were significantly associated with the willingness to accept the COVID-19 vaccine (33.9 ± 18.4 vs 9.2 ± 16.2, p < 0.001). Scores were significantly higher for males (33.7 ± 18.5 vs 27.8 ± 20.6, p < 0.001). In terms of practice settings, scores differed significantly between settings, with those in hospital pharmacies having significantly lower scores than those in community pharmacies (28.5 ± 20.2 vs 33.4 ± 18.7, p < 0.001) and those in polyclinics (28.5 ± 20.2 vs 40.1 ± 13.4, p < 0.001). There were no statistically significant differences in age, academic qualifications, year of registration or professional role.

On subjecting the behavioral determinant items to PCA, the KMO (0.87) and Bartlett test of sphericity (p < 0.001) confirmed the factorability of the correlation matrix. Five components were retained on the basis of eigenvalues exceeding 1.0 and inspection of the scree plot, explaining a total of 57.5% of the variance. The components were labelled as follows: beliefs of consequences (Cronbach's alpha = 0.81); influences of self and close others (Cronbach's alpha = 0.81); emotions (Cronbach's alpha = 0.79); influences of self and colleagues (Cronbach's alpha = 0.55); and intentions (Cronbach's alpha = 0.68) (Table 2).

**Table 2:** Responses to behavioral determinant items relating to vaccination (n = 927)

Component 1: Beliefs of consequences	Strongly disagree, n (%)	Disagree, n (%)	Neutral, n (%)	Agree, n (%)	Strongly agree, n (%)
I intend to get vaccinated because it will protect me and my family	7 (0.8)	22 (2.4)	108 (11.7)	305 (32.9)	485 (52.3)
My previous experience of receiving other vaccination such as influenza was good	10 (1.1)	59 (6.4)	174 (18.8)	374 (40.3)	310 (33.4)
I intend to get vaccinated because it will protect others in the community	8 (0.9)	22 (2.4)	83 (9.0)	350 (37.8)	464 (50.1)
It is important for me to get vaccinated because I have direct contact with patients	8 (0.9)	42 (4.5)	82 (8.8)	346 (37.3)	449 (48.4)
In Qatar, it is easy for me to get vaccinated	10 (1.1)	18 (1.9)	102 (11.0)	336 (36.2)	461 (49.7)
I believe that if I get vaccinated, it will encourage my friends and fellow colleagues to receive vaccine	9 (1.0)	39 (4.2)	136 (14.7)	447 (48.2)	296 (31.9)
Cronbach’s alpha 0.81 Mean (SD) = 61 ± 29.9 (scale -100 to 100)					

Component 2: Influences of self and close others	Strongly disagree, n (%)	Disagree, n (%)	Neutral, n (%)	Agree, n (%)	Strongly agree, n (%)
*I do not wish to get vaccinated due to religious or cultural concerns	310 (33.4)	375 (40.5)	114 (12.3)	73 (7.9)	55 (5.9)
*I will receive the COVID-19 vaccine only if it has been advised by my doctor	115 (12.4)	387 (41.7)	199 (21.5)	163 (17.6)	63 (6.8)
*I do not believe in vaccination	291 (31.4)	442 (47.7)	115 (12.4)	44 (4.7)	35 (3.8)
*I will take the vaccine only if my friends and family take it	184 (19.8)	463 (49.9)	143 (15.4)	103 (11.1)	34 (3.7)
*negatively scored Cronbach’s alpha 0.81 Mean (SD) = 24.7 ± 27.9 (scale −100 to 100)					

Component 3: Emotions	Strongly disagree, n (%)	Disagree, n (%)	Neutral, n (%)	Agree, n (%)	Strongly agree, n (%)
*I am worried about the speed of development and testing of COVID-19 vaccine	48 (5.2)	208 (22.4)	275 (29.7)	297 (32.0)	99 (10.7)
*I am not satisfied with the evidence behind the safety of COVID-19 vaccines	71 (7.7)	283 (30.5)	300 (32.4)	192 (20.7)	80 (8.6)
*I am worried about the side effects of the novel COVID-19 vaccine	64 (6.9)	214 (23.1)	267 (28.8)	272 (29.3)	110 (11.9)
*I am worried that mRNA vaccines can change the genetic makeup of people receiving it	101 (10.9)	232 (25.0)	391 (42.2)	139 (15.0)	64 (6.9)
*negatively scored Cronbach’s alpha 0.79 Mean (SD) = -1.4 ± 42.1 (scale −100 to 100)					

Component 4: Influences of self and colleagues	Strongly disagree, n (%)	Disagree, n (%)	Neutral, n (%)	Agree, n (%)	Strongly agree, n (%)
I will receive the vaccine because it will allow me to travel	36 (3.9)	121 (13.1)	105 (11.3)	366 (39.5)	299 (32.3)
My colleagues encourage me to get vaccinated against the COVID-19	34 (3.7)	148 (16.0)	151 (16.3)	427 (46.1)	167 (18.0)
Other healthcare professionals think I should get vaccinated	15 (1.6)	55 (5.9)	186 (20.1)	428 (46.2)	243 (26.2)
Cronbach’s alpha 0.55 Mean (SD) = 38.6 ± 37.6 (scale −100 to 100)					

Component 5: Intentions	Strongly disagree, n (%)	Disagree, n (%)	Neutral, n (%)	Agree, n (%)	Strongly agree, n (%)
I intend to get vaccinated because it is mandatory	44 (4.7)	230 (24.8)	122 (13.2)	320 (34.5)	211 (22.8)
I intend to get vaccinated because majority of my co-workers are vaccinated	45 (4.9)	211 (22.8)	187 (20.2)	304 (32.8)	180 (19.4)
Cronbach’s alpha 0.68 Mean (SD) = 18.5 ± 43.9 (scale −100 to 100)					

Other than the emotions component, the mean scores were positive. Within the other components, the most negative responses were to the statements, ‘I will receive the COVID-19 vaccine only if it has been advised by my doctor’ (agree/strongly agree n = 226, 24.4%); ‘My colleagues encourage me to get vaccinated against the COVID-19’ (disagree/strongly disagree n = 182, 19.7%); and ‘I intend to get vaccinated because majority of my coworkers are vaccinated’ (disagree/strongly disagree n = 256, 27.7%). For the emotions component, the mean score (SD) score was –1.2 (42.1) (scale -100 to 100). More than two-thirds were worried about the speed of development and testing of the vaccine (agree/strongly agree n = 396, 42.7%) and were worried about the side effects of the vaccine (agree/strongly agree n = 382, 41.2%). Scores for males were significantly higher than those for females (3.32 ± 41.95 vs -7.63 ± 41.3, p < 0.001). Those willing to accept the COVID-19 vaccine had significantly higher scores for component 3 than those not willing (2.9 ± 40.8 vs –35.6 ± 35.7, p < 0.001).

### COVID-19 vaccine advocacy behavior and behavioral determinants

For vaccine advocacy, the majority (n = 799, 86.2%) agreed that it was their professional duty to encourage the public to be vaccinated against COVID-19. Most (n = 860, 92.8%) had patients or colleagues asking for information on COVID-19 vaccination, and 703 (75.8%) had previously recommended seasonal influenza vaccines. Those considering it their professional duty were more likely to be male (477 (84.2%) vs 322 (83.8%), p < 0.001) and be practicing in polyclinics compared to community and hospital comparison (49 (96.1%) vs 342 (89.3%) vs 408 (82.8%), p < 0.001).

The mean percentage (standard deviation) advocacy determinant index was 36.5 ± 28.2 (scale -100 to 100), with an internal consistency of 0.85. Advocacy index scores were significantly associated with the willingness to accept the COVID-19 vaccine (42.1 ± 25.7 vs 28.1 ± 29.6, p < 0.001). Scores were significantly higher for males (40.8 ± 26.3 vs 30.9 ± 29.5, p < 0.001) and for those with more professional experience (1–5 years (6.3 ± 40.5), 6–10 years (5.9 ± 45.6), 11–15 years (–1.1 ± 45.5) (p = 0.05). Scores were significantly lower for pharmacy technicians than for clinical pharmacists or clinical pharmacy specialists (–0.3 ± 44.9 vs 14.8 vs 38.9, p < 0.05).

On subjecting the behavioral determinant items to PCA, the KMO (0.9) and Bartlett test of sphericity (p < 0.001) confirmed the factorability of the correlation matrix. Two components were retained: professional capabilities (Cronbach's alpha = 0.90) and professional role and identity (Cronbach's alpha = 0.72) (Table 3). The mean (SD) score for professional capabilities was positive (46.2 ± 31.6) (scale –100 to 100), with the most negative responses being for the statements, ‘I have sufficient evidence to recommend others to get the COVID-19 vaccine’ (disagree/strongly disagree n = 115, 12.4%) and ‘To contain the further spread of infection, I recommend COVID-19 vaccine should be made mandatory for everyone in Qatar’ (disagree/strongly disagree n = 113, 12.2%). For the professional role and identity component, the mean score (SD) was 7.3 (43.6) (scale –100 to 100), much lower than that for professional capabilities. More than one-third were unsure if they were in a position to recommend the use of the COVID-19 vaccine (agree/strongly agree n = 291, 31.4%) and preferred to wait for some time before recommending (agree/strongly agree n = 324, 35.0%). Professional capability scores were higher among pharmacists working in the community than among those working in the hospital (33.5 ± 19.0 vs 28.5 ± 20.2, p < 0.05).

**Table 3:** Responses to behavioral determinant items relating to vaccine advocacy (n = 927)

Component 1: Professional capabilities	Strongly disagree, n (%)	Disagree, n (%)	Unsure, n (%)	Agree, n (%)	Strongly agree, n (%)	Missing, n (%)
I have sufficient evidence to recommend others to get the COVID-19 vaccine	23 (2.5)	92 (9.9)	244 (26.3)	363 (39.2)	151 (16.3)	54 (5.8)
Patients place great faith in my recommendations	4 (0.4)	19 (2.0)	202 (21.8)	495 (53.4)	153 (15.5)	54 (5.8)
I will advise my family and friends to receive COVID-19 vaccine	13 (1.4)	16 (1.7)	111 (12.0)	404 (43.6)	329 (35.5)	54 (5.8)
To contain the further spread of infection, I recommend COVID-19 vaccine should be made mandatory for everyone in Qatar	35 (3.8)	78 (8.4)	141 (15.2)	302 (32.6)	317 (34.2)	54 (5.8)
I believe that my recommendations will result in higher vaccine acceptance rates	7 (0.8)	21 (2.3)	186 (20.1)	417 (45.0)	242 (26.1)	54 (5.8)
Vaccine advocacy through education is an important step to support vaccine coverage to general public	3 (0.3)	9 (1.0)	86 (9.3)	463 (49.9)	312 (33.7)	54 (5.8)
I have sufficient knowledge to advocate the use of COVID-19 vaccine	6 (0.6)	51 (5.5)	203 (22.1)	422 (45.5)	189 (20.4)	54 (5.8)
I have the necessary experience to advocate the use COVID-19 vaccines	4 (0.4)	73 (7.9)	245 (26.4)	389 (42.0)	162 (17.5)	54 (5.8)
I am confident in my ability to influence patients to receive COVID-19 vaccine	5 (0.5)	31 (3.3)	170 (18.3)	446 (48.1)	221 (23.8)	54 (5.8)
Cronbach’s alpha 0.90 Mean (SD) = 46.2 ± 31.6 (scale − 100 to 100)						

Component 2: Professional role and identity	Strongly disagree, n (%)	Disagree, n (%)	Unsure, n (%)	Agree, n (%)	Strongly agree, n (%)	Missing, n (%)
*I do not think patients will consider my advice to receive COVID-19 vaccine	88 (9.5)	320 (34.5)	286 (30.9)	123 (13.3)	56 (6.0)	54 (5.8)
*I will wait for some more time before I recommend the use of COVID-19 vaccine	59 (6.4)	312 (33.7)	178 (19.2)	254 (27.4)	70 (7.6)	54 (5.8)
*I am not sure if I am in a position to recommend the use of COVID-19 vaccine	71 (7.7)	308 (33.2)	203 (21.9)	221 (23.8)	70 (7.6)	54 (5.8)
*negatively scored Cronbach’s alpha 0.72 Mean (SD) =7.3±43.6 (scale − 100 to 100)						

## Discussion

### Statement of key findings

Almost all respondents were willing to receive the COVID-19 vaccine, with males and those based in polyclinics being more accepting. Notably, those not willing to receive the COVID-19 vaccine were more likely to have refused other vaccines in the past. Behavioral determinant index scores for acceptance were associated with behavior and were higher for males and those in polyclinics. Of the five PCA components, the emotions component had the lowest score, with scores again being associated with the behavior. Female respondents scored significantly lower in this component than males.

The majority of respondents considered it their professional duty to advocate for COVID-19 vaccination, with males and those based in polyclinics being more positive. The advocacy behavioral determinant index scores were associated with the behavior of accepting the vaccine and were higher for males. Of the 2 PCA components, the score was lowest for professional role and identity and was significantly lower for those based in hospitals.

### Interpretation

Vaccine hesitancy is a complex behavior influenced by many individual and contextual factors [9–11]. When this relates to health professionals, it may also impact their advocacy roles, as illustrated in this study with a clear relationship between not accepting the COVID vaccine and determinants relating to advocacy. The findings of this study on vaccine acceptance and advocacy and the related determinants are important given the association with past vaccine behaviour and hence are likely to also be mirrored in any future vaccine programs.

While the review of Crawshaw et al., [12] mapping questionnaire responses of similar studies to TDF, identified belief of consequences, and social and professional role and identity as key determinants, respondents in the current study were more positive in these areas. The lowest scoring PCA component reflected the emotions domain of TDF. A recent study using the Health Belief Model in Turkish pharmacists identified perceived susceptibility, severity and benefits as strong predictors of vaccine acceptance [41]. These differences may be due to several reasons, including the timing of studies in relation to the evidence base of efficacy, effectiveness and safety and the specific population studies (i.e., pharmacy professionals based in the Middle East).

The gender differences identified in this study have been reported by others [42–44], with females being more hesitant and cautious in their approach. One further study suggested that this may be influenced by safety concerns in those of childbearing age [45]. The setting-related differences (i.e., those in polyclinics most likely to be vaccinated and advocate) may be reflective of the nature of their work and patient contact. In Qatar, hospital clinical pharmacy practice is developing at a pace that is still less than that in Western countries, while in the community [46], most interactions focus on non-prescription medicines.

Complex behaviour of hesitancy will require complex interventions, as defined by the UK MRC [16]. Such interventions are more likely to be successful if developed using a theoretical basis compared to those developed more pragmatically. Behavior change interventions have been defined as `coordinated sets of activities designed to change specified behavior patterns.’ Such interventions are complex by nature and consist of several interacting components. These behavior change techniques (BCTs) are `observable and replicable components designed to change behavior'[47]. To aid intervention development, specific evidence based BCTs have been mapped to TDF domains [48]. In terms of vaccine acceptance, the TDF domain of emotions generated the lowest scores in the determinant index and hence could form the basis of the intervention. Of note, the emotion scores were statistically associated with vaccine acceptance. There were specific worries around the evidence base, speed of vaccine development, impact on genetic makeup and adverse reactions more generally. Specific BCTs mapped to emotions are advising on ways to reduce negative emotions, framing and reframing by suggesting the deliberate adoption of a new perspective, and considering the anticipated regret of not receiving vaccination. This package of interventions would require further modelling prior to being subjected to the MRC framework steps of feasibility/piloting, evaluation, and implementation.

While the majority of respondents would advocate for COVID-19 vaccination, approximately 15% would not. This is important given the public health role of pharmacy professionals, their availability and the trust placed in them by the general public [49, 50], and the evidence base for their advocacy leading to increased vaccination rates [50].

A similar approach would be to adopt intervention development in relation to vaccine advocacy. In this case, the TDF target domain for intervention development was professional role and identity, with scores being significantly lower for pharmacy technicians. It may be that the pharmacy technicians themselves do not consider themselves to be of a suitable status for vaccine advocacy, a finding that requires further investigation using qualitative approaches.

Pharmacy professionals in a number of countries are acting as vaccine administrators, a role that has been accelerated during the pandemic. A situational analysis of current practice and policy COVID-19 vaccination by pharmacists across Europe highlighted that while some countries permitted pharmacists to administer vaccines, there was potential for expansion [22, 51, 52]. A recent TDF-based survey of community pharmacists and their perspectives as vaccine administrators (not focusing solely on COVID) in Qatar highlighted that developmental interventions would require targeting knowledge, skills, and beliefs of capabilities, with changes in national legislation [53].

## Strengths and weaknesses

The strengths of this study are the use of behavioral theory, the focus on outcomes of vaccine acceptance and advocacy, and the inclusion of pharmacists and pharmacy technicians. The positive association between behavioral determinants and the behaviour of vaccine acceptance and advocacy highlights the construct validity of the two indices. These questionnaires may be of value, with some modification, in studies of non-COVID vaccination.

There are, however, several limitations that should be kept in mind when interpreting the results. The response rate of less than 40% may have introduced response bias, and the responses may also have been subjected to social desirability and other biases. Furthermore, the study was conducted in specific settings in Qatar; hence, the results may not be generalizable beyond Qatar and the Middle East.

## Conclusions

This TDF-based national study of pharmacy professionals in Qatar identified high levels of COVID vaccine acceptance and advocacy. Respondents were least positive regarding emotion-related behavioural determinants for acceptance and professional role and identity for advocacy. There is potential for behavior change technique interventions focusing on these issues, which could impact the vaccine hesitancy of pharmacy professionals and others.

## Data Availability

All data and materials essential to reproduce and validate the results presented in this article are available upon request. The journal office may obtain the necessary information by contacting the corresponding author.

## Abbreviations

BCT: Behavior change techniques
DHP: Department of Healthcare Professions
HMC: Hamad Medical Corporation
KMO: Kaiser–Meyer–Olkin
MRC: Medical Research Council
PCA: Principal Components Analysis
TDF: Theoretical Domains Framework
